# Prevalence of neuropsychiatric disorders in patients with systemic lupus erythematosus in Pakistan: A systematic review and meta-analysis

**DOI:** 10.3389/fpsyt.2023.1098734

**Published:** 2023-02-02

**Authors:** Muhammad Imran Khan, Humera Qureshi, Sohail Akhtar, Suk Joo Bae, Fazal Hassan

**Affiliations:** ^1^Department of Industrial Engineering, Hanyang University, Seoul, Republic of Korea; ^2^Department of Mathematics and Statistics, The University of Haripur, Haripur, Pakistan

**Keywords:** meta-analysis, neuropsychiatric, Pakistan, random-effects, systemic lupus erythematosus

## Abstract

**Introduction:**

By conducting a systematic review and meta-analysis, we investigated the prevalence of neuropsychiatric (NP) symptoms among systemic lupus erythematosus (SLE) patients in Pakistan.

**Methods:**

In this review work, three electronic databases (Web of Science, MEDLINE, and Google Scholar) and local databases were screened for 20 years from 1 January 2002 to 30 September 2022, to identify the articles evaluating the prevalence of NP symptoms in SLE patients in Pakistan. We performed a random-effects meta-analysis to estimate the prevalence of NPSLE. Statistical heterogeneity was measured by the I2 index, and subgroup meta-analyses were used to access the statistical heterogeneity. Furthermore, meta-regression models were used to examine the associations between prevalence estimates and study characteristics of interest. Three independent authors reviewed existing studies, extracted data, and rated the qualities of selected studies. This review was registered on PROSPERO (Registration no. CRD42022361798).

**Results:**

Thirteen studies met the inclusion criteria out of the 322 studies with a total of 2,003 SLE patients for this systematic review and meta-analysis. The prevalence of NP disorders in SLE patients was estimated to be 30.42% (95% CI:18.26–44.11%), with cognitive dysfunction being the most common (31.51%; 95% CI:1.28–76.27%), followed by headache (10.22%; 95% CI: 0.00–33.43%), seizures (5.96%; 95% CI: 3.80–8.53%), psychosis (3.64%; 95% CI: 2.38–5.13%), and neuropathy is the least common (0.86%; 95% CI: 0.00–2.74%). The heterogeneity between studies was significant (*p* < 0.01). The pooled prevalence of NP disorders among SLE patients was found highest in Punjab (41.21%) and lowest in Sindh (17.60%).

**Conclusion:**

Findings from this study revealed that SLE patients have a high prevalence of NP disorders. The most common symptoms were cognitive dysfunctions, headaches, seizures, psychosis, and neuropathy. Clinicians can manage these potentially deadly and disabling diseases more effectively if they understand the incidence of each NP symptom in SLE patients. NP symptoms among SLE patients are at their peak in Pakistan; policymakers should devise preventive strategies to curb the disease.

**Systematic review registration:**

https://www.crd.york.ac.uk/prospero/display_record. php?RecordID=361798, identifier CRD42022361798.

## Introduction

Systemic lupus erythematosus (SLE) is one of the most prevalent lupus types. SLE is an autoimmune disorder with a complex pathogenesis in which an immune system attacks the body’s own tissues, causing inflammation and tissue damage in the organs affected (1–5). It can damage the brain, the skin, joints, kidneys, blood vessels, and the lungs (6). There are no clinical treatments available for SLE patients, but medical interventions and lifestyle changes can help manage their conditions. Women are more likely to have SLE than men, with a ratio of about six women to every male (7, 8). Patients tend to have a wide range of autoantibodies, which are often linked to different clinical signs and symptoms (9, 10).

Despite numerous therapy advancements and improved diagnosis techniques, SLE continues to cause significant morbidity and mortality (11, 12). Neuropsychiatric (NP) involvement in SLE patients is one of the disease’s most dangerous side effects. It can cause negative effects on quality of life and disability (13–15). NPSLE pathogenic etiologies are likely complex (16–18), with multiple pathophysiological pathways implicated. Injury to the vascular system, blood-brain barrier (BBB), and brain parenchyma causes NPSLE symptoms (19, 20). Research shows that the damage may be caused by cytokines and autoantibodies, which can have localized or widespread effects on the central nervous system (CNS). Because the BBB does not protect the peripheral nervous system (PNS), it is vulnerable to the effects of immunological complexes, circulating autoantibodies, and other inflammatory chemicals (21). The pathophysiology of NPSLE most likely involves several antibodies (22, 23). The pathophysiological implications of NPSLE autoantibodies, which are anti-neuronal antibodies, were first investigated (24–26). NPSLE can also be caused by problems with the blood vessels, such as vasculopathy, atherosclerosis, and hypercoagulability (27, 28).

Cognitive dysfunction is a well-known sign of SLE (29–31), up to 90% patients affecting (32). Patients who do not have overt NPSLE frequently complain of cognitive issues, and rigorous neuropsychological testing commonly indicates cognitive abnormalities (33, 34). Problems with working memory, attention, and executive function are common mental abnormalities in SLE (35) and are frequently related to dysfunction in frontoparietal brain areas (36). Structural brain imaging was utilized to study these cognitive abnormalities in SLE patients (37). The results revealed structural damage to white and gray matter (38) and a higher number of white matter hyperintensities (WMHs) in SLE patients compared to healthy controls (39). This structural damage has been linked to cerebrovascular accidents, particularly in individuals with aPL autoantibodies, and breaches in the BBB, which may allow autoimmune processes to harm the brain (35, 40). WMHs have also been discovered in healthy controls, but they are much higher in SLE patients (41). These additional WMHs seen in people with SLE may cause problems with how networks connect, affecting how well people think (42).

In Pakistan, NP disorders are common in the patients with SLE. The most common disorders are depressions, anxieties, and psychoses. The other disorders include seizures, dementia, and strokes. The prevalence of NP disorders in Pakistani patients with SLE has not clearly covered. The prevalence of SLE and complications are steadily increasing in Pakistan. A number of researches (43–55) have found that NP disorders were commonly observed among SLE patients in Pakistan. To our best knowledge, there exists no official countrywide survey or national health registry for NP disorders in SLE patients in Pakistan. The goal of this work is to systematically locate, select, review, summarize, and estimate pooled prevalence of NP disorders in SLE patients using existing publications from Pakistan. The findings from this study may also contribute to the development of a management policy to lower the perceived prevalence of NP disorders in patients with SLE.

## Materials and methods

This systematic review and meta-analysis was aligned with PRISMA guidelines, and the checklists were provided in the Supplementary Table 1. They were registered with PROPERO in October 2022 (with registration no. CRD42022361798).

### Data sources and searches

Three independent authors searched Medline (*via* PubMed), Web of Sciences, Google Scholar, and local databases to identify all relevant studies published up to 5 September 2022, on the prevalence of NP disorders in SLE patients in Pakistan, regardless of language restrictions. The main used keywords were as follows: “lupus,” “neuropsychiatric” or “NP,” “NPSLE,” “SLE” or “Systemic lupus erythematosus” combined with “ACR,” “American college of rheumatology,” or “American rheumatology association” or “ARA.” Reference lists of relevant studies and reviews were also checked to identify additional articles.

### Study selection

The studies were included in this meta-analysis if they fulfilled the following criteria. (1) The studies were published up to September 2022 and looked at how often NP problems happened in SLE patients. (2) The studies were either retrospective, prospective, or cross-sectional. Studies that did not address all NPSLE symptoms, provided duplicate data, were irrelevant, or were missed during the initial assessment of abstracts were excluded (e.g., case reports or review articles).

### Data extraction

This study’s authors (M.I.K., H.Q., and F.H.) worked together to create the data extraction form in Microsoft Excel. On this information extraction sheet, the initial author’s name, the year the article was published, study design, total patients, positive patients, prevalence, setting, province, sex, male percentage, the working year, mean age of the patients, and classification criteria of SLE were all listed. Finally, the reliability of deleted data files was carefully reviewed, and any discrepancies between the deleted data were resolved by close discussions between the authors.

### Study quality assessment

The risk of bias in selected studies was independently evaluated by two authors (S.A. and F.H.) through the JBI Critical Appraisal Checklist for Studies (11). Discrepancies in the scores assigned to various aspects of methodological quality assessments were resolved through debate and adjudication by a third investigator (M.I.). The quality score (ranging from 0 to 9) was assigned to each study. Each study presents a higher possibility of bias (1–3), a medium chance (4–6), or a lower possibility (7–9), based on the score it received.

### Statistical analysis

For the pooled data, a random-effects (DerSimonian/Laird) meta-analysis model was used (56, 57), assuming the heterogeneity between the studies. Pooled results were produced at 95% confidence intervals and demonstrated with forest plots. Cochrane’s *Q*-statistic was used to test whether the heterogeneity between the studies was significant, whereas I^2^-index was employed to quantify it. The significance of heterogeneity was defined by the I^2^ value more than 50% (58, 59). The prediction interval was computed to determine the range in which the genuine effect deviates from the mean. Funnel plot, Egger regression test, and Begg’s test were conducted to investigate potential publication bias (60, 61). We conducted subgroup meta-analyses according to geographical locations, seizures, psychoses, headaches, cognitive dysfunctions, and neuropathies. To further explore the heterogeneity, univariate meta-regression models were constructed to determine the relationship between the prevalence of NP disorder in patients with SLE and the characteristics studied. The covariates in the meta-regressions included the publication year, the size of the sample, the study year, and the gender. To assess the impact of missing data from various studies on overall pooled estimates, we performed a series of sensitivity analyses in which we serially removed a study from the meta-analysis. Kappa statistic was utilized to quantify the degree of inter-rater agreement between investigators (62). All analyses were done using statistical software R (version 4.2.1).

## Results

**FIGURE 1:**
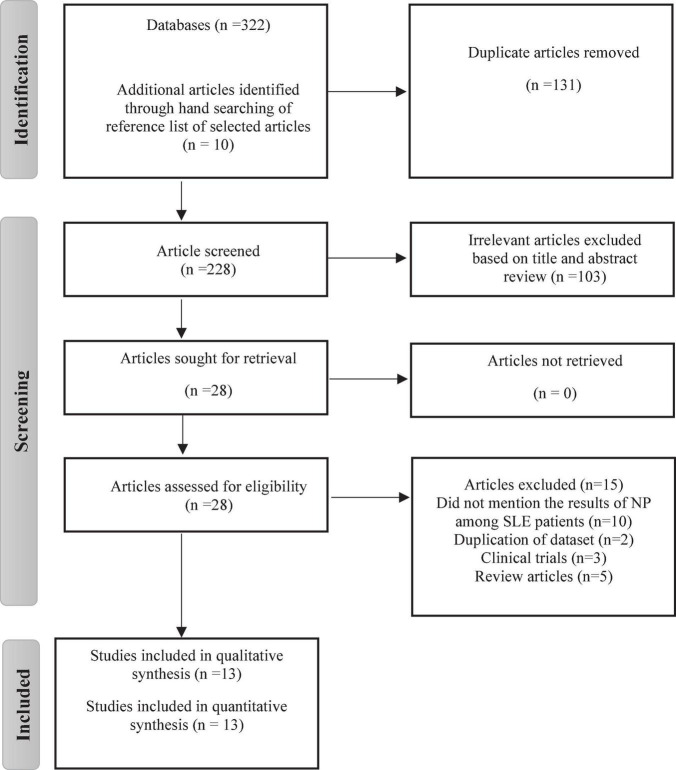
A flowchart of a PRISMA process.

Figure 1 depicts a flowchart of the PRISMA process for including or excluding articles. A total of 322 studies were found, 311 of which were found through database searches and remaining 10 articles from reference lists. After deduplication (*n* = 131), 103 studies were excluded after carefully reviewing their titles and abstracts. The remaining 28 studies were given a full-text review to determine eligibility; those failing to satisfy inclusion criteria were eliminated. Thirteen articles were finally chosen in the meta analysis. Inter-rater agreement between investigators for study selection was significant (Kappa score = 0.81, *p* < 0.01).

### Study characteristics

General characteristics of 13 eligible studies are described in Table 1. Selected studies were published between 2002 and 2022, along with 67% of the studies published in the past 10 years. It was noted that the period of participants’ inclusion was from 1986 to 2019. Ten of these studies had a cross-sectional study design (43–46, 49–53, 55) and two studies had a prospective design (47, 54), whereas one was only retrospective (48). In total, 2003 SLE patients were included. Sample sizes of SLE patients varied from 23 to 663, with an average of 103. Average ages of SLE patients were reported in all studies, ranging from 10.5 (55) to 39.99 years (51). Pooled average age of SLE patients in 13 studies was 29.86 years. The proportion of female SLE patients in 13 studies ranged from 76% (43) to 96% (51). Among 13 studies, seven studies (43, 47, 49, 51–54) were conducted in Punjab province, five in Sindh province (44–46, 48, 55), and one in Khyber Pakhtunkhwa province (50). All of the studies were based on both urban and rural areas. Nine studies had a medium risk of bias in terms of methodological quality (43–46, 48, 49, 51, 53, 54), four had a low risk (47, 50, 52, 55), and none had a high risk. Kappa score of 0.78 (*p* = 0.001) indicates that the authors agreed on the extracted data.

**TABLE 1:** Shows the general characteristics of studies chosen for the study (*n* = 13).

References	Study design	Total sample	Positive cases	Prevalence %	Province	Sex	Female %	Working year	Mean age of the patients	Bias possibility	Classification criteria of SLE
Ahmed et al. (43)	Cross-sectional	50	7	14	Punjab	Both	76	2002	22.02	Medium	ARA
Rabbani et al. (44)	Cross-sectional	196	26	13.27	Sindh	Both	87.76	1986–2021	31.09	Medium	ARA
Rabbani et al. (45)	Cross-sectional	198	29	14.65	Sindh	Both	86.87	1986–2022	31.00	Medium	ARA
Rabbani et al. (46)	Cross-sectional	198	56	28.29	Sindh	Both	87.88	1992–2005	31.09	Medium	SLICC/ACR
Raza and Khan (47)	Prospective	65	20	31	Punjab	Both	93.84	2008–2011	28.45	Low	SLICC/ACR
Ishaq et al. (48)	Retrospective	105	16	15.24	Sindh	Both	94.3	2008	31.6	Medium	ARA
Batool et al. (49)	Cross-sectional	61	40	65.57	Punjab	Both	80.3	2015–2016	26.2	Medium	ACR
Khan et al. (50)	Cross-sectional	663	202	30.47	KPK	Both	91.4	2014–2016	33.09	Low	ACR
Mumtaz et al. (51)	Cross-sectional	100	84	84	Punjab	Both	96	2016	39.99	Medium	ACR
Shamim et al. (52)	Cross-sectional	23	3	13.04	Punjab	Both	91.3	2018–2019	11.00	Low	SLICC
Butt et al. (53)	Cross-sectional	43	28	65.1	Punjab	Both	95.3	2016	28.72	Medium	SLICC
Khan et al. (54)	Prospective	269	57	21.1	Punjab	NA	NA	2018–2019	27.8	Medium	ACR
Ahmed et al. (55)	Cross-sectional	32	5	15.6	Sindh	Both	87.5	2011–2015	10.5	Low	ACR

NA, not applicable; KPK, Khyber Pakhtunkhwa; KPK province 1 study included; Sindh province 5 studies included; Punjab province 7 studies included; 10 cross-sectional studies; 1 retrospective study; 2 prospective studies; ARA, American College of Rheumatology; ACR, American College of Rheumatology; SLICC, Systemic Lupus International Collaborating Clinics.

### Quantitative synthesis

#### Pooled prevalence of NP disorders

**FIGURE 2:**
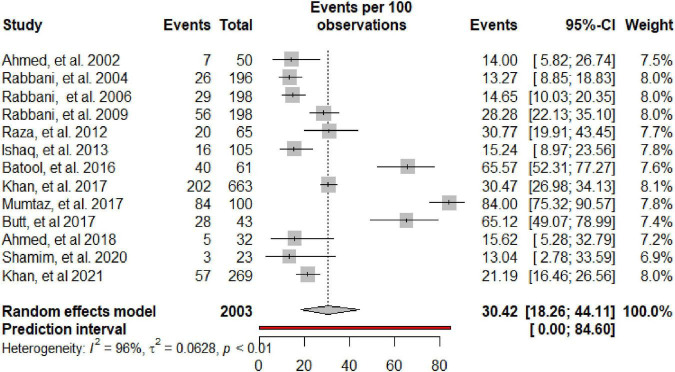
Forest plot of the prevalence of NP disorders among SLE patients in Pakistan.

**FIGURE 3:**
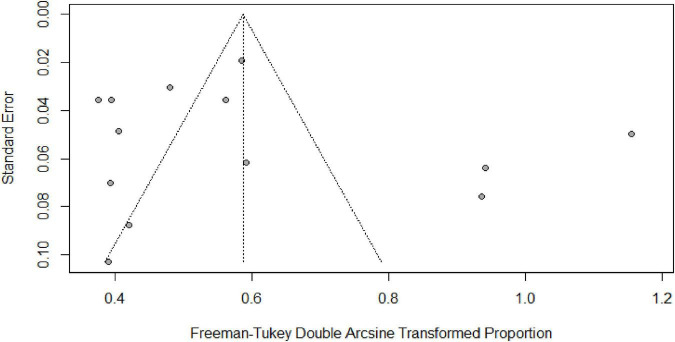
Funnel plot of the prevalence of NP disorders among SLE patients in Pakistan.

Table 2 summarizes the subgroup meta-analysis for the pooled prevalence of NP disorders in SLE patients. The prevalence of NP disorders in the SLE patients in included studies ranges from 13.04% (95% CI: 2.78–33.59%) to 84% (95% CI: 75.32–90.57%). Among SLE patients, the pooled prevalence of NP disorders was 30.42% (95% CI: 18.26–44.11%). The 95% prediction interval was 0.001 to 84.60%. The Forest plot displayed in Figure 2. The heterogeneity level in the meta-analysis was significantly high (*I*^2^ = 95.7%; *p* < 0.001). We could not find any evidence of small-study effects or publication bias based on the visual inspection of the funnel plot (Figure 3). The results of the Egger regression test (*t* = 0.53; *p* = 0.7882) and Begg’s rank test (*z* = 0.55; *p* = 0.7194) statistically support the absence of evidence for publication bias. The sensitivity analyses reveal that the pooled prevalence of NPSLE varies from 25.98% (95% CI:16.70–36.44%) to 32.14% (95% CI:19.06–46.72%) by excluding each study step by step (Supplementary Figure 1). No single study had an extreme influence on pooled NPSLE prevalence estimates.

**TABLE 2:** Summary estimates from meta-analyses of NP disorders in patients with systemic lupus erythematosus in Pakistan.

Variable	No. of articles	No. of participants	No. of cases	Prevalence, (95% CI)	*I*^2^, %	95%, prediction interval	*P*-value			
							*Q* test	Egger test	Begg test	Subgroup difference
**NPSLE**	13	2003	573	30.42 (18.26–44.11)	96	0.00–84.60	<0.001	0.7882	0.7194	
**Types**										
Seizures	8	1621	100	5.96 (3.80–8.53)	45	0.80–14.7	0.09			0.001
Psychoses	4	185	23	3.64 (2.38–5.13)	3.2	1.20–7.21	0.02			
Headaches	3	474	48	10.22 (0.00–33.43)	96.6	0.00– 100.0	0.03			
Cognitive dysfunctions	3	208	72	31.51 (1.28–76.27)	97.4	0.00–100.00	0.001			
Neuropathies	3	39	4	0.86 (0.00–2.74)	38.9	0.00; 45.93	0.1949			
**By location**										
Punjab	7	299	154	41.42 (20.14– 64.46)	97	0.00 – 100	<0.001			0.0055
Sindh	5	679	127	17.32 (11.92– 23.46)	76.8	0.00 – 55.79	<0.001			
KP	1	663	202	30.47 (26.98–34.13)						
**By age**										
Adult patients	11	1948	565	33.35 (19.38–48.96)	96.4	0.00–89.68	<0.001	0.3976	0.3487	0.0450
Pediatric patients	2	55	8	14.50 (5.99–25.47)	0.0					

#### Subgroup analysis

All subgroup analyses for the prevalence of NPSLE are shown in Table 2. The subgroup analyses show the differences in NPSLE prevalence by its disorder. Table 2 shows that cognitive dysfunctions are the most prevalent manifestation of NPSLE (31.51% CI: 1.28–76.27%), which is followed by headaches (10.22%; 95% CI: 0.00–33.43%), seizures (5.96%; 95% CI: 3.80–8.53%), psychoses (3.64%; 95% CI: 2.38–5.13%), and the least was neuropathies (0.86%; 95% CI: 0.00–2.74%). Pooled NPSLE prevalence was also found to differ by study location; the studies conducted in the Punjab province found the highest pooled prevalence estimate (41.21%; 95% CI: 6.48–81.93%), followed by Khyber Pakhtunkhwa (30.47%; 95% CI: 26.98–34.13%), and the lowest was found in Sindh 17.60% (95% CI: 8.32–29.34%). The prevalence NPSLE in significantly higher in adult population (33.35%; 95% CI: 19.38–48.96%) than pediatric population (14.50%; 95% CI: 5.99–25.47%). In the subgroup analysis, heterogeneity was high (*I*^2^ index ranged from 0.0 to 97.4%).

Using the univariate meta-regression analysis (Table 3), we observed an increasing trend with a year of study in the prevalence of NP in SLE patients. The analysis also showed that age of SLE patient is significantly correlated with the prevalence of NP disorders in SLE patients. The results showed no statistically significant relationship between the prevalence of NP disorders in SLE patients and the year of publication, the percentage of females in the sample, diagnostic method, methodological quality or the sample size of the studies.

**TABLE 3:** Univariate meta-regression analyses.

Variable	Beta (β)	*P*-value	95% CI	*R*^2^ %
Publication year	0.0159	0.1672	−0.0067– 0.0384	8.22
Year of investigation	0.0177	0.0490	0.0001–0.0353	23.02
Female ratio	−0.0002	0.3371	−0.001– 0.0007	0.00
Sample size	0.0028	0.0588	−0.0001– 0.0056	11.43
Diagnostic method	0.1518	0.1965	−0.0722–0.4652	8.99
Methodological quality	0.1225	0.44	−0.1894–0.4343	0.00
Age of SLE patient	0.0143	0.0939	−0.0024–0.0310	13.48

## Discussion

We performed, to the best of our knowledge, the first systematic review and meta-analysis on the prevalence and risk factors associated with NP disorders among SLE patients in Pakistan, based on available data published from January 2002 to September 2022. The study used the data from 13 unique data sets with 2003 SLE patients from geographically diverse populations of Pakistan. Our study is purposed to provide useful information about the creation of public health measures to reduce NP disorders in SLE patients. Pooled overall prevalence of NP among SLE patients was 30.1%, indicating that approximately one out of every three SLE patients living in Pakistan is suffering from NP disorders. The findings of this meta-analysis are in line with the recent meta-analysis conducted in the Swiss lupus cohort study (28.1%) (63). However, pooled overall prevalence of NPSLE in Pakistan is significantly lower than in the studies conducted in Switzerland at 56.3% (64) and Egypt at 50.7% (65). This discrepancy could be attributed to the differences in research methodology, sample size, and universal definition of NPSLE disorders.

In our meta-analysis, as in many other studies, headaches, cognitive dysfunctions, psychoses, and seizure were the most frequent neurological disorder (66–68). The subgroup analyses show that cognitive dysfunctions was the most common NP manifestation, affecting 31.51% of SLE patients. The results are somewhat similar with another meta-analysis which showed that 39% prevalence of cognitive dysfunctions in SLE patients (33). Some studies have reported the results that persons with SLE have a greater, although extremely varied, prevalence of cognitive dysfunctions ranging from 17 to 66% (33, 69). In part, the disparities are attributed to a lack of a universal cognitive definition in many existing studies.

The subgroup analysis showed that pooled prevalence of NP disorders in SLE patients significantly varies with geographical location. The highest pooled prevalence of NP disorders was found in Punjab province at 41.42%, which was followed by Khyber Pakhtunkhwa province (30.45%) and Sindh province (17.32%). The wide disparity in prevalence between studies is due to variations in geographical location, ethnicity, sample bias, study design screening methodologies, the terminology used to define the event, the lack of specificity of NP symptoms, and the extent to which the occurrence is linked to SLE (70–72).

Admittedly, our systematic review and meta-analysis study has the following limitations. First, most of studies (69%) included in the meta-analysis had medium risk of bais and only four studies had a low risk of bias. Second, the results of the meta-analysis are only based on the data from three provinces. We have not found any articles from Baluchistan and Azad Kashmir. Even though these are the most populous provinces in the country, we should be careful in the generalization of the results to entire country. Thirdly, we limited our search to peer-reviewed studies and excluded gray literature, which may lead to some publication bias in our study. Fourth, in the included studies, we found a high level of heterogeneity in our analysis, which is commonly observed in meta-analyses of prevalence data (73, 74). This showed that of the variability in NPSLE prevalence measurements is due to the heterogeneity between the studies as opposed to chance. This is because that NPSLE is not a single disease entity (75), but rather a mixture of diverse disorders with potentially distinct pathophysiologic processes, including the production of autoantibodies (76). None of the ACR’s 19 NP syndromes are specific to SLE; they have been reported in association with systemic vasculitides, antiphospholipid syndrome, Sjogren’s syndrome, Behcet’s disease, rheumatoid arthritis, and many other autoimmune disorders, as well as in individuals without autoimmune disease (77–80).

Even though there are some limitations, this is the first systematic study and meta-analysis to investigate how common NP disorders are in SLE patients in Pakistan as a whole. Before we started the study, we published the protocol of the study that explained how we would do it. We also used scientific and statistical methods to gather and analyze the data. Different subgroup analyses and random effects meta-regression analyses were conducted to assess numerous variables that could influence our estimates. Despite the high heterogeneity, this systematic review and meta-analysis still provides useful and important information for the pooled prevalence of NP disorders in SLE patients in Pakistan. As conducting high-quality primary research on the prevalence of NP in SLE patients is often very expensive, and it can take years until the findings can finally be analysed.

## Conclusion

This study provides pooled estimates of NP disorder among SLE patients in Pakistan. The figures suggest that NPSLE is a significant public health issue in Pakistan. Over the last several decades, there has been an uptick in the overall prevalence of NP symptoms in the general population in Pakistan. This upward trend is likely to continue in the foreseeable future. Since NP symptoms among SLE patients in Pakistan are on the rise, the government of Pakistan needs to work on developing an NPSLE preventative strategy and control programs that can be implemented across the entire country. Furthermore, there is a significant variation in the prevalence of NPSLE in different provinces of Pakisan. Therefore, a countrywide study is recommended on pathogenesis of NP disorder in SLE patients in Pakistan, and to find the relative prevalence of each symptom relative to matched controls, such as individuals with other autoimmune disorders or apparently healthy subjects.

## Data availability statement

The original contributions presented in this study are included in the article/Supplementary material, further inquiries can be directed to the corresponding authors.

## Author contributions

All authors listed have made a substantial, direct, and intellectual contribution to the work, and approved it for publication.
