# Supplementary material for: Prevalence of neuropsychiatric disorders in patients with systemic lupus erythematosus in Pakistan: A systematic review and meta-analysis
Source: Front Psychiatry. 2023 Feb 2;14:1098734. doi: 10.3389/fpsyt.2023.1098734 (PMC9931908; doi:10.3389/fpsyt.2023.1098734)
Supplement: Supplementary file 1 [file Data_Sheet_1.docx]

**Supplementary File**


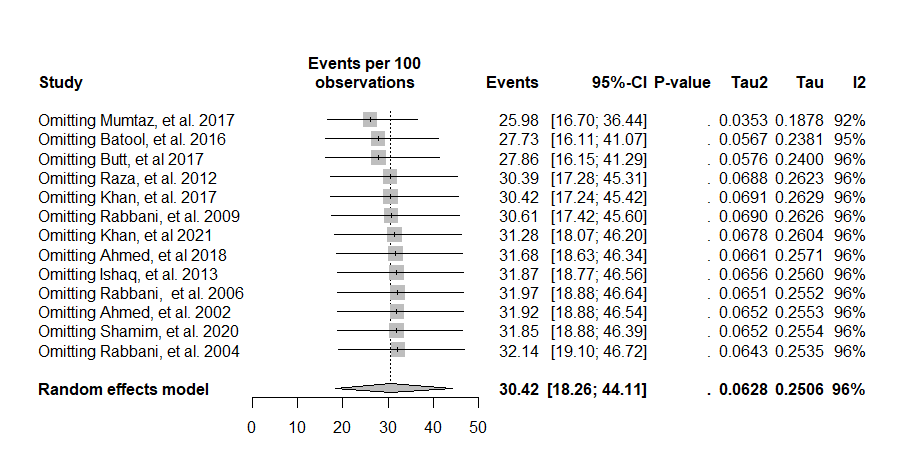


**sFigure 1**: Forest plot of the sensitivity analysis for the prevalence of NPSLE in Pakistan
